# 

**Published:** 1915-04-10

**Authors:** 


### Buildings Contemplated and in Progress.

Alton.—The Guardians have purchased land, at a cost of £1,000, as a site for the proposed infirmary.

Ballinasloe.—Tenders are invited for the erection of two temporary blocks for male and female patients, each block to accommodate fifty patients, at the District Asylum, Ballinasloe. The buildings are to be erected in the Hy-Rib system of reinforced concrete. Specifications may be obtained of Mr. G. B. Meenan, 83 Botanic Road, Dublin.

Drumshoreland.—The Midlothian County Council are considering a proposal to lease a site from the Drumshoreland Hospital Committee for the erection of a number of shelters and a hospital pending the completion of the sanatorium scheme.

Dudley.—The work of preparing the site of the new eye infirmary and out-patient department of the Guest Hospital has been commenced. The site adjoins the hospital grounds and faces Tipton Road. The contract for the building amounts to £4,050.

Gravesend.—The Town Council are considering the provision of additional isolation hospital accommodation.

Huntingdon.—The Hants County Council have instructed the Public Health Committee to get plans prepared for a combined sanatorium and hospital.

Portsmouth.—The Guardians have decided to apply to the Local Government Board for sanction to borrow £2,000 for the enlargement of the Nurses' Home.

Portsmouth.—The Corporation of Portsmouth invite tenders for the erection of a tuberculosis hospital at Langstone, Portsmouth. Particulars may be obtained of Mr. A. E. Tutte, architect, 38 London Road, North End, Portsmouth.

Wakefield.—The Guardians have decided to carry out extensions and alterations to the workhouse from plans prepared by Messrs. Simpson and Firth.

Warwick.—The Warwickshire King Edward VII. Memorial Committee have approved plans for the memorial sanatorium proposed to be erected on Hertford Hill, near Warwick. The plans are by Mr. West, architect, of London.

Watford.—The Guardians have decided to build a nursery at the workhouse at an estimated cost of £3,530.

### Gifts and Bequests.

Mrs. Helen McLarty Birkmyre, of Broadstone, Port Glasgow, N.B., left £5,000 to Broadstone Jubilee Hospital, Port Glasgow.

The treasurer of Ancoats Hospital, Manchester, has received the sum of £250, being the legacy left by the late Mr. S. Megarity.

At the recent annual meeting of governors of Leicester Royal Infirmary opportunity was taken by the Palace Matinée Committee to hand to the chairman a cheque for £206 4s., as the proceeds of the recent matinée at the Palace Theatre. This was the most successful of several similar efforts, and acknowledgment was paid by Mr. Gee, chairman of the board, to Mr. Oswald Stoll, Mr. A. Baum, and Mr. G. Somerville, the chairman and treasurer of the local committee, and to Messrs. J. H. Ward and Trueman Towers, the honorary secretaries, for their valuable co-operation.

Mr. William Richmond, coachbuilder, whose estate has been provisionally valued at £18,255, has left his residence and grounds at Lancaster to the Royal Lancaster Infirmary, and, subject to certain devises of real estate and legacies to relatives and friends, directed that the net residue of his estate shall be paid over to the trustees of the infirmary for founding, endowing, and maintaining a convalescent home in connection with the institution, to be called the Richmond Convalescent Home.

### Doctors' Wills.

Mr. Thomas David Ransford, F.R.C.S., L.R.C.P., of Limpley Stoke, Bath, late of Queen Square, honorary surgeon to the Royal United Hospital, Bath, formerly surgeon to the Royal Southern Hospital, Liverpool, and honorary medical officer to the Liverpool Dispensaries, has left estate of gross value £16,467.

The sum of £1,460 Consols has been transferred to the trustees of the Nelson Hospital, Wimbledon, by the executors of the late Mrs. Jane Davis, in respect of a legacy of £1,000 free of duty.

### Medical Appointments.

DR. WILLIAM ANGUS, formerly of Hereford, has been appointed acting medical officer of health for the City of Leeds, at a salary of £600 a year, to fill the vacancy caused by the retirement of Dr. Spottiswoode Cameron.

DR. ROBERT BRUCE LOW, M.R.C.S., L.R.C.P., has been engaged as *locum tenens* at Lambeth Infirmary until the end of May, at £6 6s. a week, when he leaves for Singapore; whilst Dr. G. S. Miller has arranged to stay a few weeks longer as third medical officer.

### Personalia.

IT is proposed to extend the appointment of Dr. Edward Squire, C.B., as expert medical adviser to the London Insurance Committee until the end of June. The Committee has under consideration at the present time the question of making arrangements on a permanent basis for this work.

MR. OSCAR WARBURG has resigned the position of chairman of the Medical Service Sub-Committee of the London Insurance Committee, which he has held since its formation. The resignation has been accepted with regret, and Mr. H. E. A. Cotton has been appointed to succeed him.

AT a special meeting of the board of governors of the Leicester Royal Infirmary, Mr. H. Simpson Gee has been re-elected chairman and Mr. C. J. Bond vice-chairman for the ensuing year. A hearty welcome was given to Mr. S. F. Stone, the treasurer, on resuming his attendance after a long absence through illness. Mr. J. D. Cradock, of Quorn, was elected a member of the board in the place of Mr. E. H. Warner, resigned.

### The Book Market.

LIST OF NEW BOOKS RECEIVED FROM THE FOLLOWING PUBLISHERS:—

### Pamphlets and Papers.

Simpkin, Marshall and Co., Ltd.: "Hospital Handbook in English and French." By H. Meugens. 1s. net.

"Organisation and Management of Auxiliary Classes." By Helen MacMurchy, M.D., Inspector of Auxiliary Classes for Ontario.

J. M. Dent and Sons: "A Plea for the Thorough and Unbiassed Investigation of Christian Science." By Charles Hennan Lea. 1s. net.

"Femur Fractures: Statistics of End-Results." By John B. Walker, M.D. Reprinted from the *American Journal of Surgery*. And "Fracture of the Neck of the Femur: its Treatment." Reprinted from the *N.Y. State Journal of Medicine*.

### Institutional and Public Reports.

Pontypool and District Hospital: Report for 1914.

Aberdeen Royal Mental Hospital: Annual Report for 1914.

Transactions of the Incorporated Sanitary Association of Scotland, 1914.

The Meath Home, Godalming: Annual Report for 1914.

King Edward VII. Hospital, Cardiff: Annual Report for 1914.

"About Cremation": The Cremation Society of England, 324 Regent Street, W.; and Report for 1914.

Royal Hospital for Diseases of the Chest, City Road, E.C.: Report for 1914.

University of London, University College: Report for 1914.

Blackburn and East Lancashire: Report for 1914.

Barry Infirmary: Annual Report for 1914.

British Hospitals Association: Report of the Council for 1914

Rates of Subscription: Twelve months, 6/6; Six months, 4/-; Three months, 2/-, post free to any address in the United Kingdom. Abroad, Twelve months, 8/8; Six months, 5/-; Three months, 2/6, post free.—Address The Subscription Dept., "The Hospital," The Hospital Building, 28/29 Southampton Street, Strand, London, W.C.

